# Supplementary material for: Reactive metabolite production is a targetable liability of glycolytic metabolism in lung cancer
Source: Nat Commun. 2019 Dec 6;10:5604. doi: 10.1038/s41467-019-13419-4 (PMC6898239; doi:10.1038/s41467-019-13419-4)
Supplement: Supplementary file 1 — Supplementary Information [file 41467_2019_13419_MOESM1_ESM.pdf]

Supplementary Information

Reactive metabolite production is a targetable liability of glycolytic metabolism in lung cancer

Luengo et al.

Supplementary Fig. 1

a

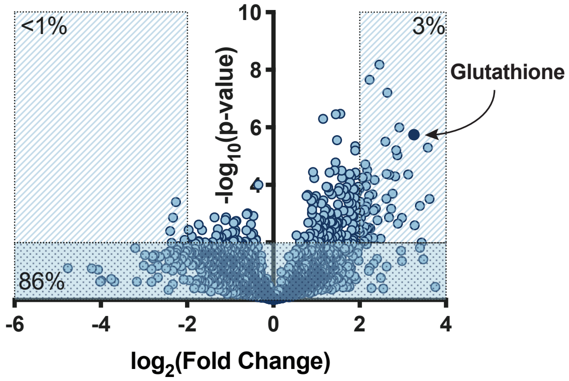

d

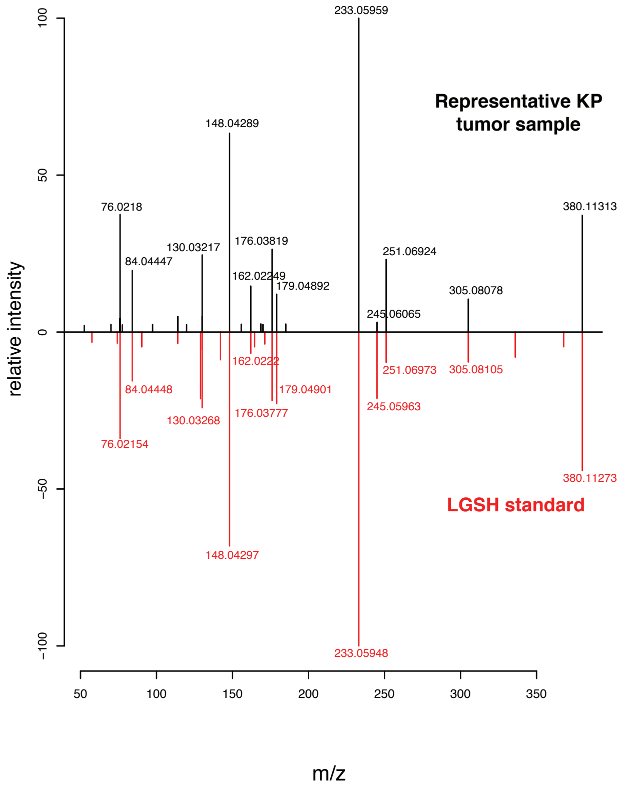

b

Glutathione adducts

	Description	$-\log_{10}$ (p-value)
GS-CHO	S-Formylglutathione	4.1
GS-$C_3H_5O_2$	S-D-Lactoylglutathione	3.8
GS-C_2H_2O	S-(Formylmethyl)glutathione	3.8
GS-CH_2O	S-hydroxymethylglutathione	3.4
GS-$C_4H_4O_4$	Succinated glutathione (GSF)	3.3

c

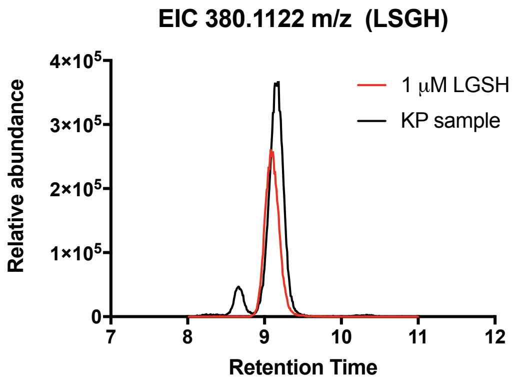

e

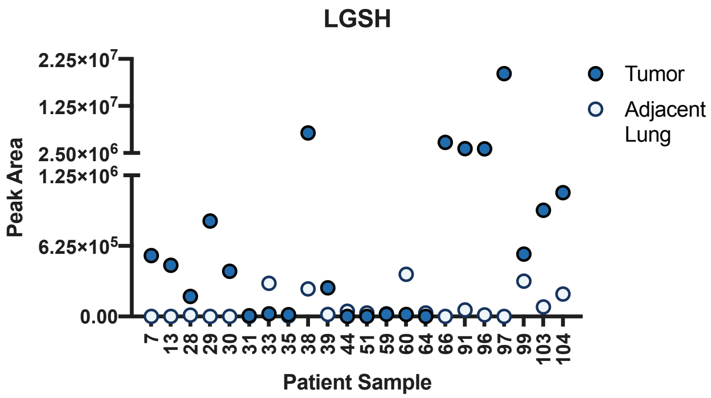

Supplementary Figure 1. Evidence for increased glutathione adducts in lung tumor tissue.

(a) Volcano plot indicating metabolites changing in mouse lung tumor tissue relative to normal lung tissue as determined by untargeted LCMS analysis. Lung tumors were generated using the LA2 or the KP NSCLC mouse models. Most metabolites were unchanged between tumor and normal tissue (86%), while 3% were significantly increased in tumor tissue and <1% were significantly decreased. Glutathione is indicated with an arrow. Peaks with fewer than an average of 5×10^4 ion counts were excluded from the analysis.

(b) Glutathione adducts detected by untargeted LCMS analysis of tissue extracts with levels that were significantly higher in mouse lung tumor tissue compared to normal lung tissue are shown. The mouse lung tumors analyzed were generated using the LA2 and KP models.

(c) Overlay of extracted ion chromatograms (EIC) for m/z 380.1122 [+H] in a pure lactoylglutathione (LGSH) standard (red) and a representative biological sample (from a KP lung tumors)(black). For the biological sample, only the 9 minute peak was integrated for analysis.

(d) Mirror plot showing an MS/MS spectrum collected from a pure LGSH standard (bottom; red) aligned with an MS/MS spectrum from the corresponding LGSH peak in a representative biological sample (from a KP lung tumor)(top; black).

(e) Relative LGSH levels detected in matched lung tumor and adjacent non-cancer lung tissue samples resected from 22 human NSCLC patients as determined by LCMS.

Supplementary Figure 2

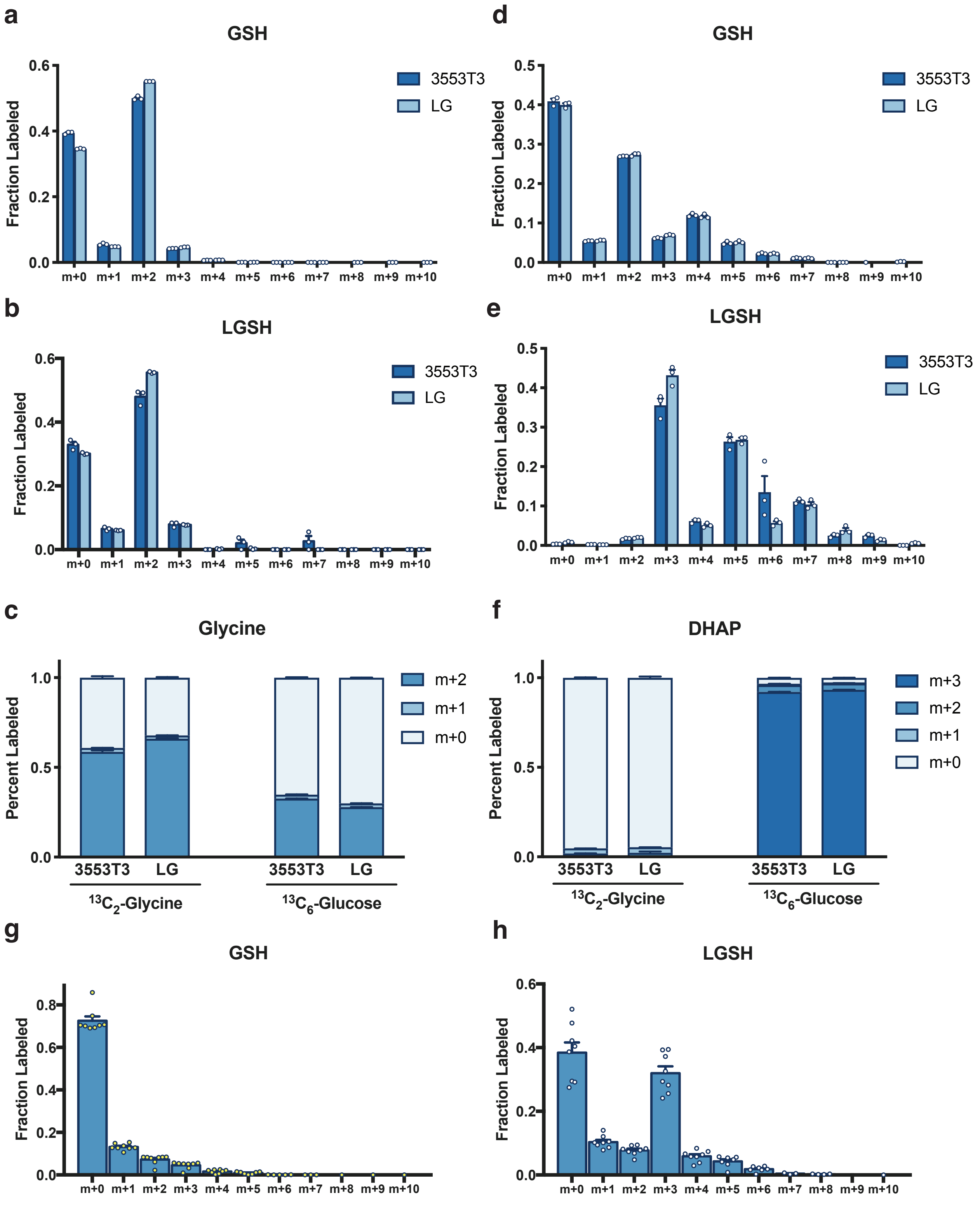

Supplementary Figure 2. Methylglyoxal is produced as a byproduct of glycolysis in NSCLC.

(a and d) Isotopomer distribution of reduced glutathione (GSH) as measured by LCMS in 3553T3 and LGSP (LG) lung cancer cell lines independently derived from lung tumors arising in KP mice that were cultured for 24 hours in the presence of [U-$^{13}C_2$]glycine (a) or [U-$^{13}C_6$]glucose (d) (n=3).

(b and e) Isotopomer distribution of lactoylglutathione (LGSH) as measured by LCMS in 3553T3 and LGSP (LG) lung cancer cells cultured for 24 hours in the presence of [U-$^{13}C_2$]glycine (b) or [U-$^{13}C_6$]glucose (e) (n=3).

(c and f) Isotopomer distribution of glycine (c) and the glycolytic intermediate dihydroxyacetone phosphate (DHAP, f) was measured by LCMS in 3553T3 and LGSP (LG) lung cancer cells cultured for 24 hours in [U-$^{13}C_2$]glycine or [U-$^{13}C_6$]glucose as indicated.

(g and h) Isotopomer distribution of GSH and LGSH as measured by LCMS in autochthonous lung tumors arising in LA2 mice following a 6 hour infusion with [U-$^{13}C_6$]glucose. Values were normalized to plasma enrichment of glucose (n = 8).

Values in all panels indicate mean ± SEM

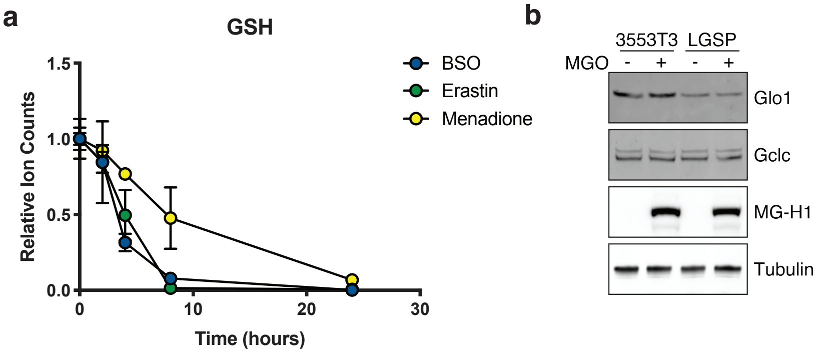

Supplementary Figure 3. Glutathione depletion sensitizes NSCLC cells to methylglyoxal.

(a) LCMS quantification of reduced glutathione (GSH) in 3553T3 NSCLC cells that had been treated with 100 µM buthionine sulfoximine (BSO), 2 µM erastin, or 10 µM menadione for the indicated amount of time. The peak area was normalized to the protein content of each sample and to the initial time point. Values indicate mean ± SEM (n=3).

(b) Western blot analysis to assess expression of glyoxalase I (Glo1), glutamate-cysteine ligase catalytic subunit (GCLC), and proteins containing the MG-H1 epitope in 3553T3 and LGSP lung cancer cells that had been cultured in the absence or presence of 200 µM methyglyoxal for 24 hours as indicated.

Supplementary Fig. 4

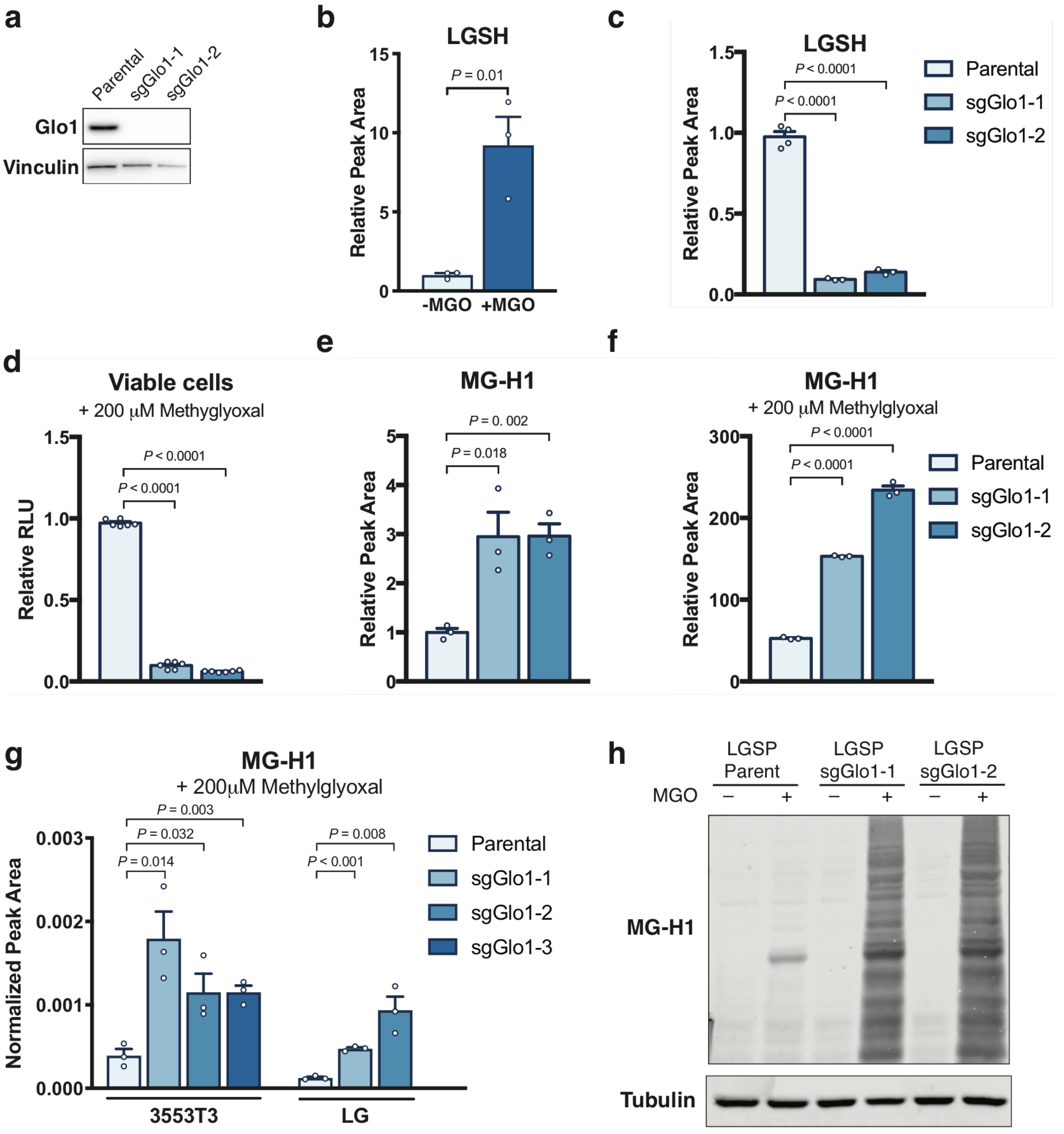

Supplementary Figure 4. Glyoxalase 1 is required for methylglyoxal detoxification

(a) Western blot analysis assessing glyoxalase I (Glo1) expression in parental LGSP lung cancer cells and in independent clones where the Glo1 gene was disrupted using CRISPR/Cas9 (sgGlo1) as indicated.

(b) Relative levels of lactoylglutathione (LGSH) in 3553T3 parental NSCLC cells that had been incubated in the presence or absence of 200 μM methylglyoxal (MGO) as indicated (n=3).

(c) Relative levels of LGSH as detected using LCMS in LGSP parental cells or the Glo1-deleted clones described in (a). The peak area of LGSH is normalized to the peak area of GSH (n=3).

(d) Relative number of viable LGSP parental cells and Glo1-deleted clones as determined by CellTiter-Glo Luminescence Assay following a 48-hour incubation with 200 μM methylglyoxal (n = 3).

(e) LCMS quantification of relative free MG-H1 abundance in LGSP parental cells and in Glo1-deleted clones. Values shown are the peak area of MG-H1 normalized to the peak area of arginine (n = 3).

(f) LCMS quantification of relative free MG-H1 abundance in LGSP parental cells or in Glo1-deleted clones following incubation with 200 μM methylglyoxal for 16 hours. Values are normalized to MG-H1 peak area in parental cells cultured in the absence of methylglyoxal (and shown in panel e), which were analyzed in the same LCMS experiment (n=3).

(g) LCMS quantification of free MG-H1 abundance in 3553T3 and LGSP parental lung cancer cells or in the Glo1-deleted clones (described in Fig. 4a) following incubation with 200 μM methylglyoxal for 16 hours. The peak area measured for each sample was normalized to the protein content and to a deuterium-labeled internal MG-H1 standard, which was added to the extraction solvent at a concentration of 100 nM.

(h) Western blot analysis assessing proteins containing MG-H1 epitopes in lysates from LGSP parental cells and in Glo1-deleted clones that had been treated without or with 200 μM methylglyoxal for 12 hours.

All *P* values were calculated by unpaired, two-tailed Student's t-test. Values in panels b, c, d, e f, and g indicate mean ± SEM.

Supplementary Fig. 5

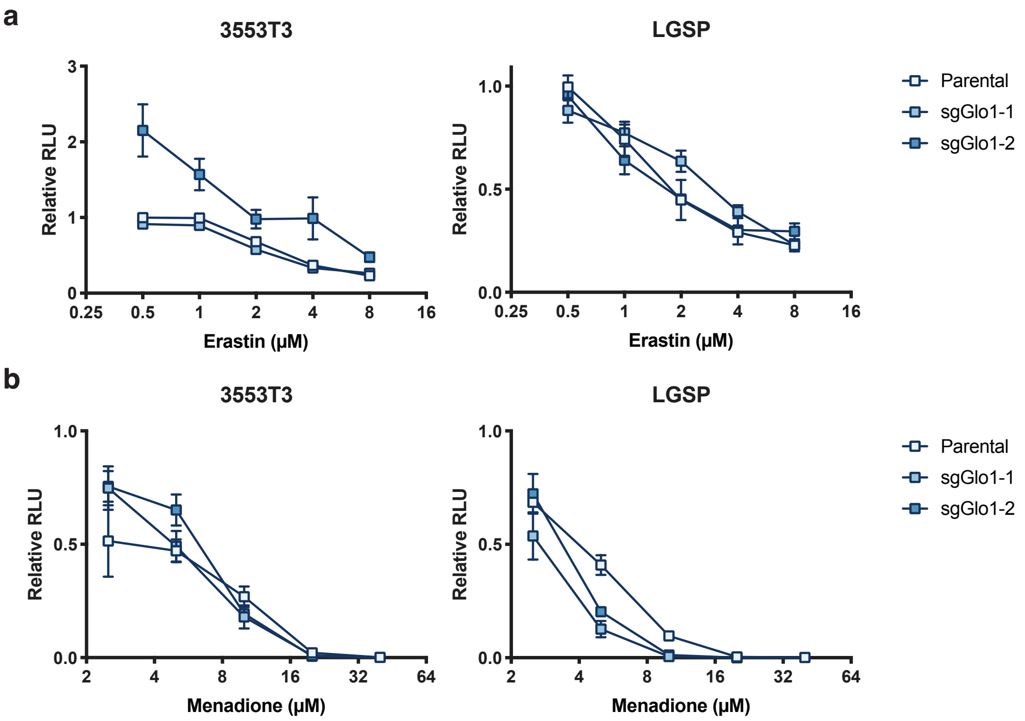

Supplementary Figure 5. Glyoxalase 1 deletion does not alter sensitivity to erastin or menadione.

The effect of the indicated concentration of erastin (a) and menadione (b) on cell viability was assessed using the CellTiter-Glo Luminescence Assay in 3553T3 and LGSP parental cells and Glo1-deleted clones as indicated. Values in all panels indicate mean ± SD (n=3).

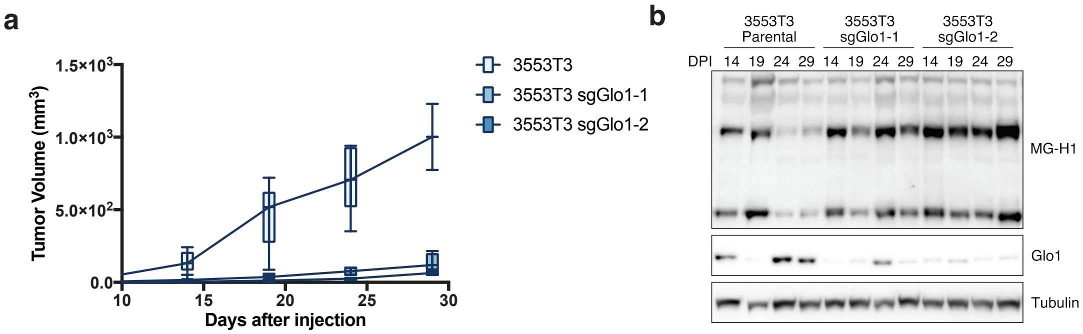

Supplementary Figure 6. Assessment of MG-H1 accumulation in NSCLC tumors over time

(a) Tumor growth of allografts generated from parental 3553T3 cells and Glo1-null clones from 10-30 days post-injection (DPI) is shown. The tumor volume for each genotype is depicted as a box and whisker plot for each time point. The difference in tumor growth between parental cell lines and Glo1-deleted clones was significant for every comparison ($P < 0.0001$ by two-way ANOVA; n = 12).

(b) Samples were collected from the tumors shown in (a) every 5 days starting at 14 days post-injection (DPI) and Western blot analysis was performed on lysates to assess Glo1 expression and accumulation of proteins containing MG-H1 epitopes over time.

Supplementary Figure 7

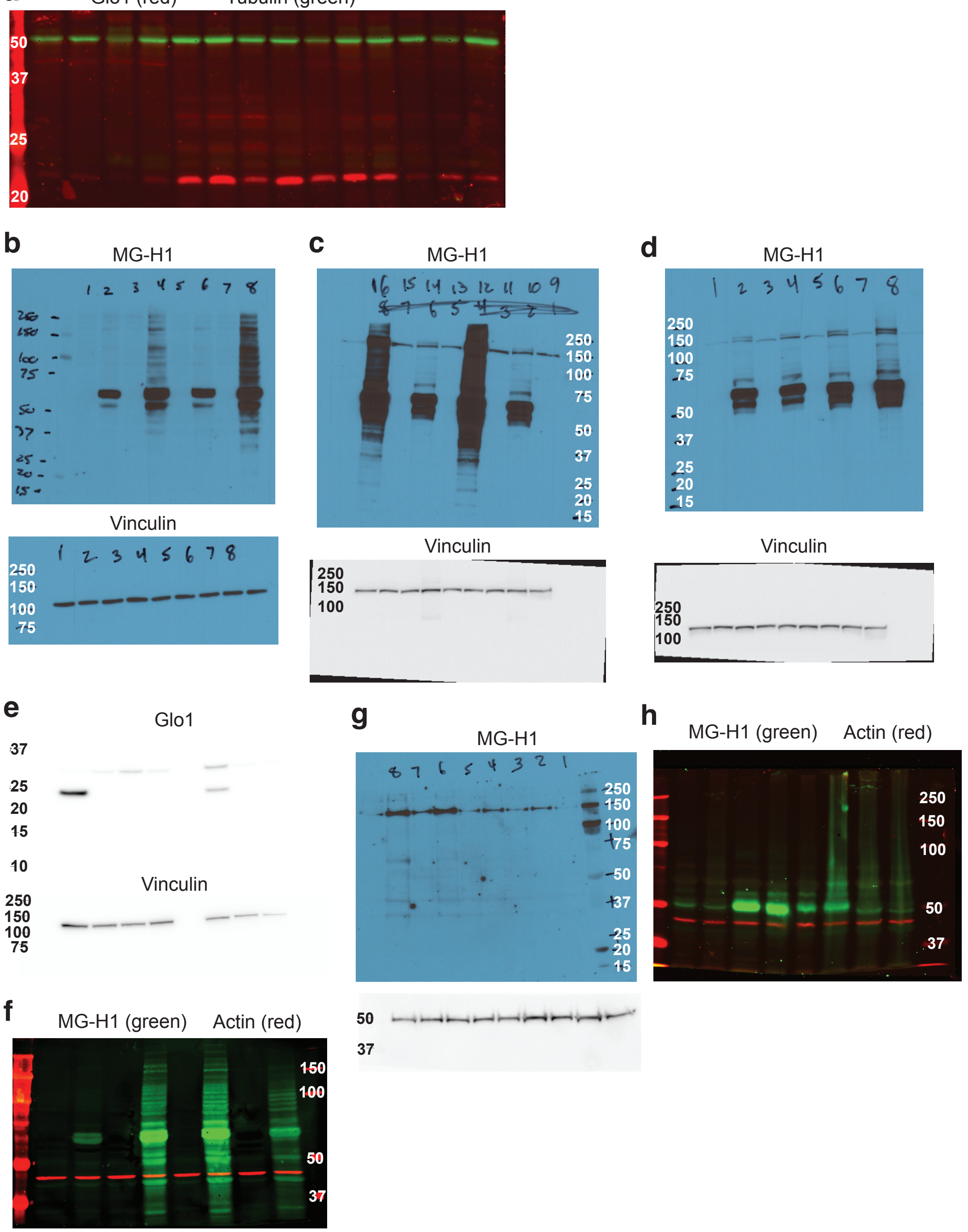

Supplementary Figure 7. Uncropped figures from western blots

Uncropped western blot images that correspond to Figure 1f (a), Figure 3e (b), Figure 3f (c), Figure 3g (d), Figure 4a and Supplementary Figure 4a (e), Figure 4f (f), Figure 5a (g), and Figure 5d (h).

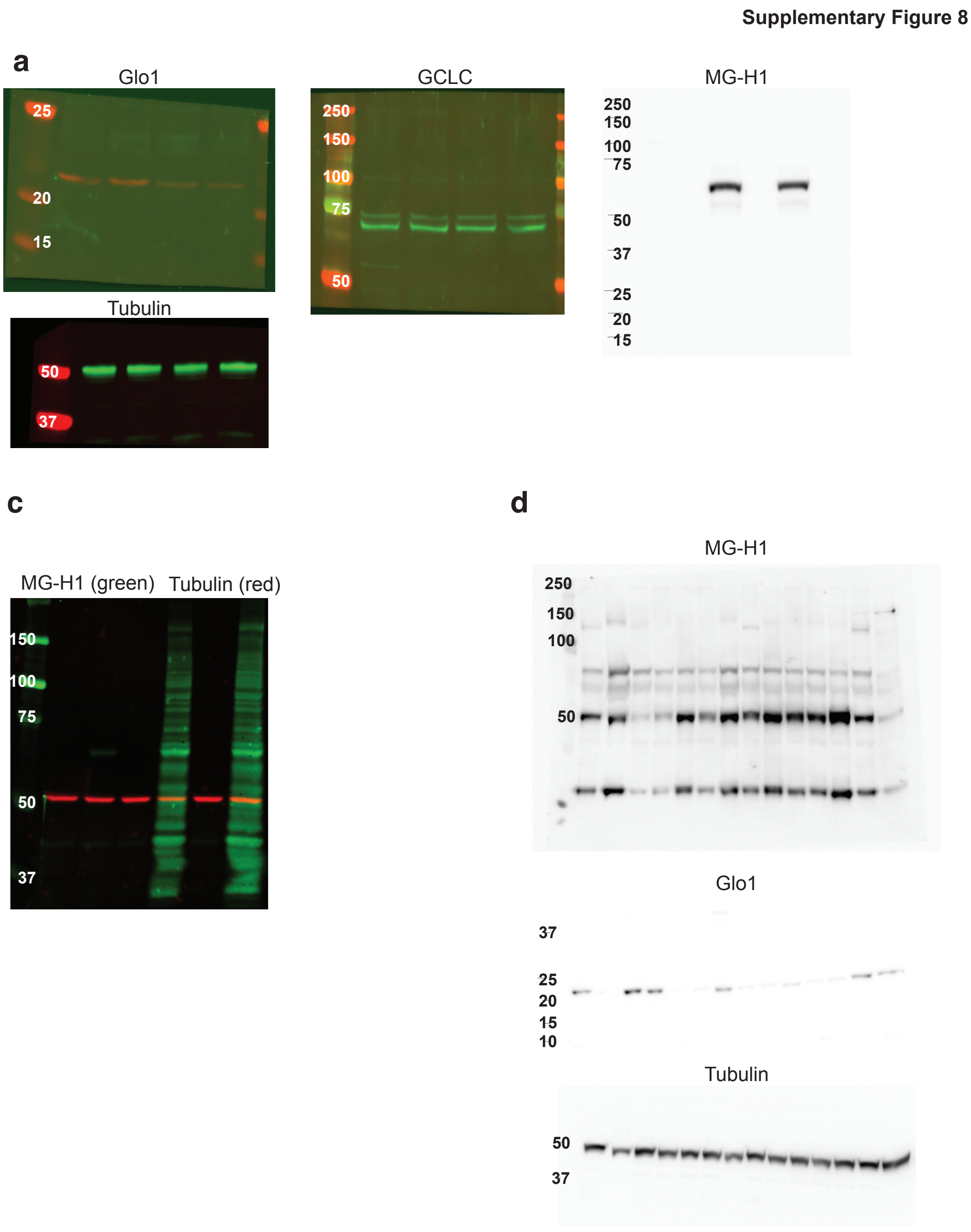

Supplementary Figure 8. Additional Uncropped figures from Western Blots

Uncropped western blot images that correspond to Supplementary Figure 3b (a), Supplementary Figure 4h (b) and Supplementary Figure 6b (d).
